# Tobacco policy coverage in California jurisdictions before and after enactment of proposition 56

**DOI:** 10.1016/j.pmedr.2025.103080

**Published:** 2025-04-25

**Authors:** Dennis R. Trinidad, Candice D. Donaldson, Brian Dang, Matthew D. Stone, Thet Nwe Myo Khin, Sara B. McMenamin, Yuyan Shi, Tam D. Vuong, Xueying Zhang, Karen Messer, John P. Pierce

**Affiliations:** a Herbert Wertheim School of Public Health and Human Longevity Science, University of California, San Diego, USA; b Moores Cancer Center, University of California, San Diego, USA; c California Tobacco Prevention Program, California Department of Public Health, Sacramento, CA, USA; d Office of Population Health, University of California Davis Comprehensive Cancer Center, Sacramento, CA, USA

**Keywords:** Tobacco, Flavored tobacco, Multi-unit housing, Policy, Secondhand smoke, Geographic areas, Population studies

## Abstract

**Objective:**

Proposition 56, a $2 tobacco tax enacted in California in 2016, led to increased funding to Local Lead Agencies which work to reduce tobacco use. We examined whether Proposition 56 was associated with increases in the population covered by local policies addressing four areas: 1) tobacco retail sales (TRS), 2) flavored tobacco products sales (FTP), 3) outdoor secondhand smoke (SHS) restrictions, and 4) smoking restrictions in multi-unit housing (MUH).

**Methods:**

2007–2023 data from the Policy Evaluation Tracking System in California were analyzed. The unit of analysis was the California jurisdiction, with outcome the time (in months) to policy enactment in a jurisdiction. Kaplan-Meier estimates and population coverage percentages were calculated by weighing each jurisdiction by its population size. Discrete-time survival models were fitted to test the effect of Proposition 56 on the rate of population coverage for each policy of interest.

**Results:**

By January 2023, 79 % of the California population was covered by a local SHS policy but only 55 %, 47 % and 18 % was covered by a local TRS, FTP and MUH policy, respectively. The rate of increase in TRS and FTP policy coverage was greater post-Proposition 56 than pre-Proposition 56 (*p* < 0.001), while the rate of increase did not change significantly for MUH and SHS policies.

**Conclusions:**

Proposition 56 was associated with marked increases in the enactment of TRS and FTP, but not SHS or MUH policies. Despite increases post-Proposition 56, additional efforts are needed to increase local adoption of TRS, FTP and MUH policies because coverage remains low.

## Highlights

• California enacted Proposition 56 ($2 tobacco tax) to reduce tobacco use in 2016.
• Increased adoption of tobacco retail sales policies since Proposition 56 enactment.
• Increased adoption of flavored tobacco sales policies since Proposition 56 passed.
• No impact on adoption of smoke-free multi-unit housing policies post-Proposition 56.

## 1 Introduction

Cigarette smoking has been declining across the US since the 1980s (Leas et al., 2023). In California, this decline has been more rapid than in other states due to its active state-level tobacco control program (Pierce et al., 1998a), which has also led to declines in lung cancer mortality (Pierce et al., 2019). In 2016, California voters approved Proposition 56, which increased tobacco taxes by $2/pack. This was the first increase in state tobacco taxes since 1999. The revenue generated from Proposition 56 was allocated to various state health programs and entities, including the California Tobacco Prevention Program (CTPP). California's program has been one of the few that emphasize tobacco control action at the local level as evidenced by the recent documentation of nearly 400 US local government ordinances on tobacco flavorings, with approximately 80 % having occurred in either California or Massachusetts (Campaign for Tobacco-Free Kids., 2025). The CTPP was allocated funds from Proposition 56 starting in 2018, leading to a substantial increase in tobacco control funding to Local Lead Agencies (LLAs) (California Tobacco Prevention Program, 2024). CTPP has also funded an enhanced monitoring program for local ordinances that does not exist in other states. In this paper, we use this monitoring program to describe the association of funding with the passage of laws in California.

For more than 30 years, CTPP has utilized an approach that includes engaging local governments to assist in changing social norms around tobacco use to help reduce the availability and consumption of tobacco products across the state (Roeseler and Burns, 2010) with a goal of ending the tobacco epidemic and eliminating tobacco-related health inequities (*California Tobacco Facts and Figures*, 2024; Lee et al., 2015; Contributors, 2016). Key CTPP priorities are to further reduce exposure to secondhand smoke, especially in identified problem areas such as outdoor settings and multi-unit housing, and to reduce the effectiveness of tobacco industry marketing to populations that use tobacco at higher rates. These priorities can also be operationalized as moral social norms (Anderson, 2014) as there is widespread agreement that smokers do not have the right to expose non-smokers to the dangers of secondhand smoke (World Health Organization, 2025) A key CTPP strategy has been to encourage local action to enforce these rights (*Bonta R California Continues Investment to Combat the Illegal Sale of Tobacco Products to Youth: Attorney General Announces New Funding Available for Local Governments Through the* 2024-2025, 2025). The California Cigarette and Tobacco Products Licensing Act in section 22971.3 specifically states that the state law does not preempt local laws except for the collection of state taxes (*Casetext I. Division 8.6 - Cigarette and Tobacco Products Licensing Act of* 2003, 2024).

CTPP supports community-level tobacco prevention efforts by funding local health departments and tobacco prevention programs, or LLAs, that serve the 61 local health jurisdictions (58 counties and three cities) in California (California Department of Public Health, 2024). LLAs aid in efforts to prevent and reduce tobacco use through the development and implementation of jurisdiction-level policy interventions (California Department of Public Health, 2024). In California, local policy jurisdictions typically refer to city, county, or special districts with the authority to enact and enforce local laws within its geographical boundaries (Public Health Law Center, 2015). LLAs serve as backbone agencies (California Department of Public Health, 2021) within their health local jurisdiction by working to advance policy and implement stricter local tobacco control regulations than those set by the state (Policy Evaluation Tracking System, 2023).

CTPP works with LLAs on tailored comprehensive tobacco prevention plans. CTPP provides state assistance via an online forum where LLAs can discuss plans with other LLAs and CTPP grantees, as well as providing model ordinances that can be adapted to their local jurisdiction(s) as well as technical assistance (California Department of Public Health, 2021). CTPP tracks local policy coverage progress covering four key areas: i) tobacco retail sales restrictions; ii) restricting the availability of flavored tobacco products; iii) smoke-and-vape-free multi-unit housing; and iv) reducing secondhand smoke in outdoor settings.

While prior studies have examined the impact of Proposition 56 on tobacco use outcomes, including prevalence (Keeler et al., 2020) and product pricing (Gunadi et al., 2021), there is a lack of research on the relationship between Proposition 56 and changes in the enactment of these four local jurisdiction-level policies. With the influx of funds from Proposition 56 to support tobacco control efforts, we hypothesize that the enactment of each of these policies will increase at a greater rate after Proposition 56. We examine changes in the enactment of each of the above-mentioned policies across California jurisdictions before and after the 2018 allocation of Proposition 56 funds to the CTPP.

## 2 Methods

### 2.1 Data source

The current study utilized CTPP's Policy Evaluation Tracking System (PETS) (California Department of Public Health/California Tobacco Prevention Program, 2024) to evaluate changes in local policy jurisdiction coverage before and after the allocation of Proposition 56 funds to CTPP and other CTPP-funded partners. PETS is a policy surveillance database that collects and scores ordinances enacted within local Californian jurisdictions. This project is a secondary data analysis of public use files and does not involve contact with human subjects and thus meets the criteria for exemption from human subjects protection review by the Institutional Review Board at University of California San Diego.

Policy details are collected through a variety of avenues including: (1) daily scans of tobacco policy news sources, such as the Tobacco.org, Google News, government databases, internet search engines, and social media; (2) posting quarterly solicitation requests for information on newly adopted or amended ordinances on California tobacco control websites; (3) local reporting of pending or new policies on monthly CTPP Community of Practice calls; (4) county coalition meetings/emails/newsletters, and (5) contact with local clerks for relevant supplemental policy information. Policies enacted in all 539 California jurisdictions were documented by qualified coders using a set of scoring rubrics developed for each policy type (California Department of Public Health/California Tobacco Prevention Program, 2024) and the enactment date of the policy was recorded. Policy enactment dates ranged from 2007 to 2023 and the most recent enactment date was used for a given policy type. The policy indicators described below refer to the enactment of any policy in each jurisdiction, regardless of policy strength or comprehensiveness.

We note that jurisdictional policy coverage examined in this report is in addition to any state-level laws that were enacted over time. However, it is important to track jurisdiction-level policy coverage because state tobacco laws may not be enacted, monitored or enforced consistently at the local level; and jurisdiction policies may be more stringent than state law.

### 2.2 California population and jurisdictions

California, with a population of 39.1 million in 2023, is divided into 58 county jurisdictions. Within each county, municipalities can incorporate and have their own elected representatives (Wikipedia, 2024). Thus, in addition to county jurisdictions, there were a total of 481 incorporated city jurisdictions in the PETS data. The populations of these cities and counties are estimated from the American Community Survey (ACS), an ongoing monthly survey of the US Census Bureau that uses an addressed-based sample and completes ∼3.5 million surveys/year. Response rates to this survey average well over 80 % (US Census Bureau, 2024). The ACS was utilized for population size estimates to use as frequency weights in our statistical model, described below. Five-year population estimates from 2016 to 2020 form the basis for estimating policy coverage for the state and across jurisdictions.

### 2.3 Outcome definitions

#### 2.3.1 Tobacco retail sales policies

A jurisdiction is considered to have a tobacco retail sales policy if it enacted any policy that restricts free samples, discounts on tobacco products, and the sale of tobacco or electronic smoking devices. This includes policies establishing tobacco-free pharmacies, minimum price or minimum pack size, and limits the number of retail licenses a jurisdiction can have based on population density or proximity to schools and residential areas.

#### 2.3.2 Flavored tobacco policies

A jurisdiction is defined to have a flavored tobacco policy if it enacted any policy that restricts the sale of flavored tobacco products at the local level (e.g., restricting the sale of menthol cigarettes and e-cigarettes/vapes). Such policies are in addition to state-level laws or propositions with similar goals, often with local efforts and policies preceding state-level laws, and with local laws often being more robust than state laws (American Lung Association, 2024). Flavored tobacco policies at the jurisdictional level may be stricter than state law by including additional restrictions, such as limiting the sale of flavored tobacco products near schools (i.e., requiring a minimum tobacco retailer distance from a school) or have a broader definition of flavored tobacco products than the state (e.g., flavored hookah, flavored loose-leaf tobacco).

#### 2.3.3 Multi-unit housing

A jurisdiction is defined to have a multi-unit housing policy if it enacted any policy that established a minimum of two units be free from combustible tobacco smoke (e.g., cigarettes, cigars) (Public Health Law Center, 2025). Such policies may also include e-cigarettes/vapes or smoking or vaping marijuana. However, vape-only or marijuana-only policies were not counted as having a multi-unit policy.

#### 2.3.4 Secondhand smoking policy

A jurisdiction is defined to have an outdoor secondhand smoking policy if it enacted any policy that restricts cigarette smoking in any of the following: outdoor dining areas, outdoor bar areas, outdoor public events and venues, outdoor recreation areas, outdoor transit stops/shelters, outdoor public easements/rights of way, or outdoor worksites.

### 2.4 Statistical analysis

The unit of analysis is California jurisdictions and the primary outcome measure is the time it takes, in months, for a jurisdiction to enact a given policy. Two time intervals, pre-Proposition 56 funding (January 2007–January 2018) and post-Proposition 56 funding (February 2018 – January 2023) were used in this analysis. The PETS dataset was expanded using the ‘survSplit’ function of the ‘survival’ package so each jurisdiction would have two pseudo-observations, one for each time interval, and a time-varying indicator function for the post-Proposition 56 period was created (Therneau, 2023). An exposure time variable was created to denote the total number of months until a jurisdiction enacts a policy. For example, if a jurisdiction enacted a policy in January 2019, the pre-Proposition 56 observation for this jurisdiction would have an exposure time of 132 months (2007–2018) since it did not enact a policy in this time interval. The post-Proposition 56 observation for this jurisdiction would have an exposure time of 12, since it took 12 months into the post-Proposition 56 period before a policy was enacted.

To test the effect of Proposition 56 on the rate of population coverage, a discrete-time survival model was fitted for each of the four policies (a log linear regression model with outcome the number of jurisdictions adopting the policy in a given month, a quasipoission link function, and an offset of log exposure time in months, using the ‘glm’ function in R). Coefficient *t*-tests and analysis-of-variance omnibus F-tests were run to test the overall significance of model terms (Hastie & Pregibon, 2025) at 5 % significance. Jurisdiction coverage percentages were calculated using Kaplan-Meier estimates and population coverage percentages were found by weighing each jurisdiction by their population size. All analyses were performed using R version 4.3.3.

## 3 Results

### 3.1 Jurisdiction coverage of policies over time

**Fig. 1:**
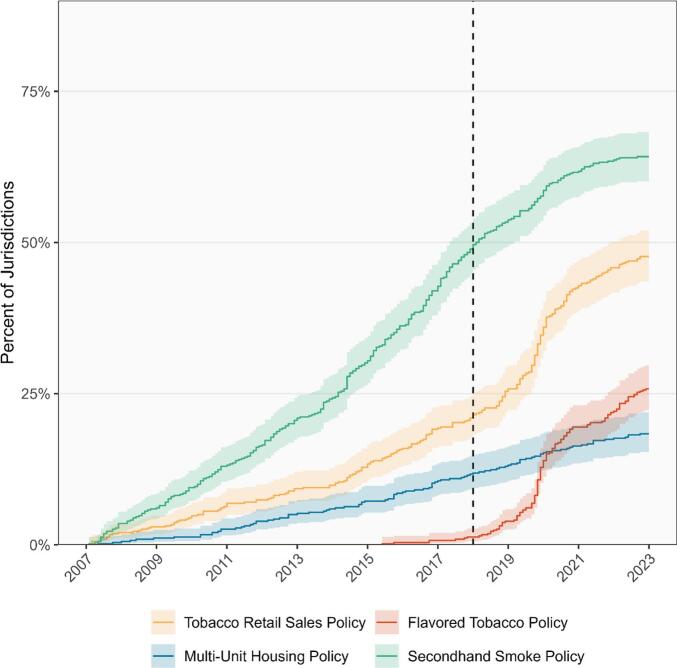
Percentage of California Jurisdictions Covered by Each of Four Tobacco Policies of Interest, 2007–2023. Notes Tobacco Retail Sales Policy refers to local policies that restrict free samples, discounts on tobacco products, and the sale of tobacco or electronic smoking devices. Flavored Tobacco Policy refers to local policies that restrict the sale of flavored tobacco products. Multi-Unit Housing Policy refers to local policies that require a minimum of two units be free from combustible tobacco smoke. Secondhand Smoke Policy refers to local policies that restrict smoking in public places.

Fig. 1 illustrates time trends in jurisdiction coverage for each of the four policies of interest since the first jurisdiction enacted a policy in 2007 until the last date examined in 2023. By 2018, approximately 20 % of California jurisdictions had a tobacco retail sales policy. Post-Proposition 56, the rate of adoption increased substantially such that about 47 % of jurisdictions had a policy regarding tobacco retail sales by 2023. Although the rate of adoption was greater post-Proposition 56, the dramatic increase was not evident until about two years after the bill was passed, in 2020.

Although the first jurisdictions to enact flavored tobacco policies started in mid-2015, the rate of adoption remained low with only about 2 % of jurisdictions having such a policy by 2018. The rate then increased rapidly post-Proposition 56, to about 25 % by 2023, or 4.7 percentage points each year. However, the dramatic increase in flavored tobacco sales policies was also not evident until about two years after the passing of Proposition 56.

By the passing of Proposition 56 in 2018, about 10 % of jurisdictions had multi-unit housing policies. The average rate of increase was not significantly different after the passing of Proposition 56 and by 2023 only about 15 % of jurisdictions had multi-unit housing policies.

Outdoor secondhand smoke policies were first enacted by jurisdictions in 2007 and increased wherein about 50 % of jurisdictions had such a policy by the time Proposition 56 was passed in 2018. Post-Proposition 56, the rate of increase slowed and by 2023 about 65 % of jurisdictions had secondhand smoke policies.

### 3.2 Maps of county-level policy coverage

**Fig. 2:**
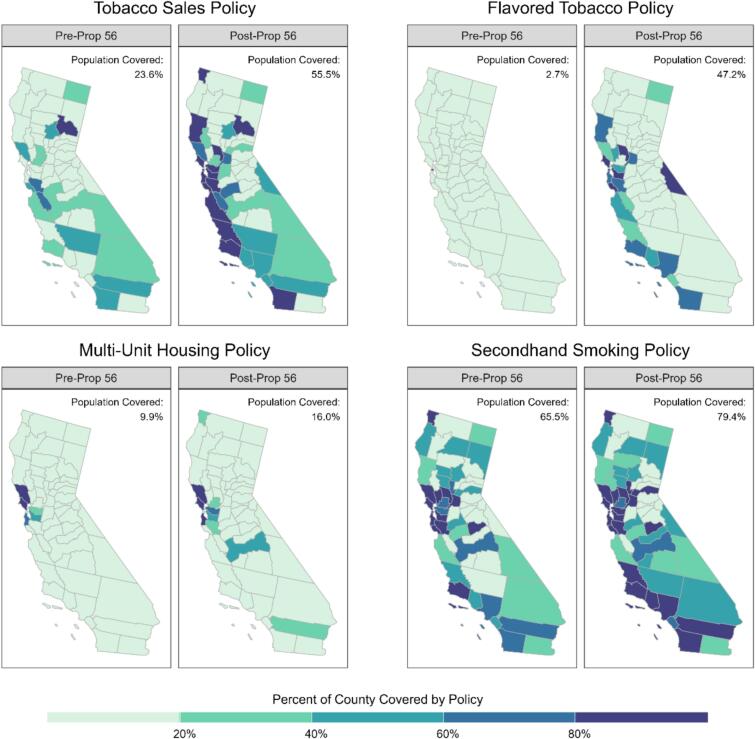
Map of California Counties Showing Coverage for Each of Four Tobacco Policies of Interest, Pre- and Post-Proposition 56, 2007–2023. Notes Pre-Prop 56: Jan. 2007 – Jan. 2018. Post-Prop 56: Feb. 2018 – Jan. 2023. Tobacco Retail Sales Policy refers to local policies that restrict free samples, discounts on tobacco products, and the sale of tobacco or electronic smoking devices. Flavored Tobacco Policy refers to local policies that restrict the sale of flavored tobacco products. Multi-Unit Housing Policy refers to local policies that require a minimum of two units be free from combustible tobacco smoke. Secondhand Smoke Policy refers to local policies that restrict smoking in public places.

Fig. 2 presents maps of California, divided into 58 counties, with changes over time in the percentage of each county's population which was covered by a local policy for each of the tobacco policies of interest. Only three counties, located in Northern California, had greater than 60 % population coverage for tobacco retail sales policies pre-Proposition 56. Post-Proposition 56, most counties along the coast had over 80 % coverage for tobacco retail sales policies.

Pre-Proposition 56, there were no counties in the state with greater than 20 % population coverage for flavored tobacco policies. After the enactment of Proposition 56, most counties along the coast had at least 20 % coverage for flavored tobacco policies with some in Southern California and the San Francisco Bay Area having more than 60 % coverage.

Pre-Proposition 56, only five counties in the state had greater than 20 % coverage for multi-unit housing policies. Of these counties, which were located in the San Francisco Bay Area, three had greater than 60 % coverage. Only five additional counties had greater than 20 % coverage for multi-unit housing policies after Proposition 56, with most located in the northern part of the state.

The majority of California counties had outdoor secondhand smoking polices prior to the enactment of Proposition 56, with many San Francisco Bay Area counties having greater than 80 % coverage. After Proposition 56, a large majority of counties were covered by outdoor secondhand smoking policies, with additional counties in Southern California having greater than 80 % coverage.

### 3.3 Discrete-time survival analyses

Table 1 summarizes the results of the discrete-time survival analysis to test whether time trends in the adoption of each policy type across jurisdictions differed before and after the 2017 passage of Proposition 56.

**Table 1:** Jurisdiction and Population Coverage of Four Tobacco Policies in California, January 2018 and January 2023 (Pre- and Post-Proposition 56 Funding), from Discrete-Time Survival Analyses.

Policy Type		Jurisdiction (%)	Jurisdiction Coverage Rate	*p*-value	Population (%)	Population Coverage Rate	p-value
Tobacco Retail Restrictions	Pre-Prop 56	21.5	ref		23.6	ref	
	Post-Prop 56	47.7	7.81	<0.01	55.5	9.14	<0.01
Flavored Tobacco Sales Restrictions	Pre-Prop 56	1.3	ref		2.7	ref	
	Post-Prop 56	25.8	15.30	<0.01	47.2	14.04	<0.01
Smoke-Free Multi-Unit Housing	Pre-Prop 56	11.7	ref		9.9	ref	
	Post-Prop 56	18.4	0.94	0.91	16.0	1.10	0.85
Secondhand Smoking Restrictions	Pre-Prop 56	49.7	ref		65.5	ref	
	Post-Prop 56	64.2	0.72	0.27	79.4	0.88	0.54

Notes:

Pre-Prop 56: % covered at the funding date Jan 1, 2018

Post-Prop 56: % covered on Jan 31, 2023

*p-values were obtained from a Wald test of significance in a Poisson regression model for discrete-time survival analysis

Tobacco Retail Sales Policy refers to local policies that restrict free samples, discounts on tobacco products, and the sale of tobacco or electronic smoking devices.

Flavored Tobacco Policy refers to local policies that restrict the sale of flavored tobacco products.

Multi-Unit Housing Policy refers to local policies that require a minimum of two units be free from combustible tobacco smoke.

Secondhand Smoke Policy refers to local policies that restrict smoking in public places.

Jurisdiction (%) refers to the percentage of city and county jurisdictions within California that enacted a particular policy.

Jurisdiction Coverage Rate refers to the rate of increase in jurisdictions enacting a policy from pre- to post-Prop 56.

Population (%) refers to the percentage of the California population that was covered by a particular policy.

Population Coverage Rate refers to the rate of increase in the California population covered a particular policy from pre- to post-Prop 56.

### 3.4 Tobacco retail sales policies

Approximately 21.5 % of jurisdictions had tobacco retail sales policies in the pre-Proposition 56 period, which increased to 47.7 % post-Proposition 56. The rate of policy adoption in the post-Proposition 56 period was significantly greater than in the pre-Proposition 56 period (*p* < 0.01). Similarly, 23.6 % of the California population was covered by tobacco retail sales policies in the pre-Proposition 56 period, which increased to 55.5 % post-Proposition 56. The rate of increase in population coverage during the post-Proposition 56 period was significantly greater than that during the pre-Proposition 56 period (p < 0.01).

### 3.5 Flavored tobacco policies

A very small percentage (1.3 %) of jurisdictions had policies restricting flavored tobacco sales in the pre-Proposition 56 period. However, this increased to 25.8 % post-Proposition 56. The rate of policy adoption across jurisdictions and the rate of increase in population covered was significantly greater in the post-Proposition 56 period than in the pre-Proposition 56 period (*p* < 0.01 for both comparisons). While only 2.7 % of the California population was covered by policies restricting flavored tobacco sales pre-Proposition 56, this increased to 47.2 % post-Proposition 56.

### 3.6 Multi-unit housing policies

In the pre-Proposition 56 period, only 11.7 % of jurisdictions had multi-unit housing policies that covered 9.9 % of the California population. While the percentage of jurisdictions with multi-unit housing policies increased to 18.4 % post-Proposition 56, representing 16 % of the population, the rates of adoption in the post-Proposition 56 period were not significantly different from the pre-Proposition 56 period (*p* < 0.80 for both comparisons).

### 3.7 Secondhand smoking policies

A substantial percentage of jurisdictions (49.7 %) had outdoor secondhand smoking policies, covering 65.5 % of the state's population, in the pre-Proposition 56 period. Although this increased to 64.2 % of jurisdictions, representing 79.4 % of the population, the rate of policy adoption in the post-Proposition 56 period was not significantly different from the pre-Proposition 56 period (*p* > 0.25 for both comparisons).

## 4 Discussion

Proposition 56 was associated with marked increases in the enactment of tobacco retail sales policies and flavored sales policies, but not in outdoor secondhand smoke or multi-unit housing policies. Thus, our hypothesis that the adoption rate of all policies would increase after Proposition 56 was only partially supported. By January 2023, about 80 % of the California population was covered by outdoor secondhand smoking policies, while only about half were covered by tobacco retail sales and local flavored tobacco policies. Less than 20 % were covered by multi-unit housing policies.

The lack of significant change in the rate of adoption of outdoor secondhand smoke policies post-Proposition 56 suggests that moral social norms regarding the protection of non-smokers from secondhand smoking were supported in many jurisdictions even before Proposition 56 and were not changed with the new law. Despite California's statewide smoke-free workplace law to protect nonsmokers from secondhand smoke (Pierce et al., 1998b), a recent review concluded that active smoking in outdoor hospitality venues exposes customers and staff even in adjacent outdoor and indoor non-smoking areas, to secondhand smoke at levels that exceed the World Health Organization's guidelines (Tong et al., 2024). Therefore, efforts must continue to ensure that as much of the state's population is protected from the harms of secondhand smoke, particularly in the inland northern regions of California.

The enactment of Proposition 56 did not appear to change the rate of adoption of multi-unit housing policies, which was slow throughout the study period of 2007–2023, such that only 16 % of the state's population had multi-unit housing coverage by January 2023. This low rate, despite enactment of Proposition 56 and the resulting increased funding to LLAs that prioritized multi-unit housing (California Tobacco Prevention Program, 2024) (as one of four focal areas), highlights difficulties with the enactment of such policies and suggests that increased allocation of efforts and resources in this tobacco control domain are needed. It has been reported that more than one in four multi-unit housing residents in the US with a current smoking status allowed smoking inside the home and supported allowing smoking inside all multi-unit housing apartments or living areas (Reyes-Guzman et al., 2023), reinforcing how multi-unit housing residents are unable to control the level of secondhand smoke or aerosol exposure incursions within their places of residence. Additionally, there is active debate related to personal freedoms within individual housing units, and imposing smoking bans may be viewed as an infringement on such freedom, making it especially challenging to garner support for multi-unit housing policies, including monitoring and enforcing such policies. Tobacco industry efforts to counter multi-unit housing policies have also worked against enactment (Miller and Vijayaraghavan, 2022). Nonetheless, California jurisdictions may benefit from examining processes undertaken in the San Francisco Bay Area as three counties in the region achieved greater than 80 % population coverage for multi-unit housing policies.

To adolescents, flavors are one of the most appealing characteristics of tobacco products and a leading reason for usage (Clodfelter et al., 2023; Kaplan et al., 2023) and most adolescents have been found to have easy access to flavored tobacco products (Clodfelter et al., 2023; Leas, 2024). Both tobacco retail sales policies and flavored policies showed marked increases in jurisdictional enactment about two years after the passing of Proposition 56, beginning approximately in 2020 (Fig. 1). This lag illustrates the length of time it takes for major policies to be enacted within jurisdictions even with substantial increases in activity and LLA funding (California Tobacco Prevention Program, 2024), which include activities such as building local capacity, the development of implementation plans, community organization and mobilization, and the securing of agreement of local policy makers (Hellesen et al., 2024). While this lag could also be related to the documented spike in youth e-cigarette/vape use between 2017 and 2019 (Pierce et al., 2023), following the aforementioned dramatic increases, rates of enactment continued an upward trend for the next two years, though at a slower rate. It is possible that the slowing of jurisdictional coverage of tobacco retail sales policies, flavored policies and outdoor secondhand smoke policies that began in 2021 may have been related to the COVID-19 pandemic, as much of the population interacted through remote or virtual means, which could have negatively affected enactment processes. Rates of jurisdictional coverage of each of these policies should continue to be tracked in the future.

### 4.1 Limitations

This study has several limitations that should be noted. Jurisdictions can have multiple policies across each of the policy areas of interest and our analyses do not account for multiple local policy enactments or how the passing of a particular policy (e.g., Tobacco 21) affected the passing of another (e.g., retail sales policies). Similarly, local level policies may have interacted with state-wide laws that had similar goals, though it should be noted that local efforts and policies often precede state-level laws. Although state-wide smoke-free policies do not preempt local ordinances, we also acknowledge that the delay in implementing the state-wide law on flavors while awaiting referendum results may have led some local jurisdictions to defer a local ordinance. Another limitation is that the PETS data only assessed whether a policy was enacted or not. Finally, as we used a database that was unique to California, we were unable to obtain similar information on a comparison group. Thus, the associations reported may not generalize well to other states.

## 5 Conclusions

Despite these limitations, the findings presented here should be informative to prevention scientists, practitioners, and policy makers as they indicate that much work remains to be done to increase local adoption of tobacco retail sales policies and flavored sales policies. Despite an increase in the rate of adoption post-Proposition 56, only about half of California's population was covered by a policy in each of these two areas. The enactment of multi-unit housing policies is a significant challenge as it has the lowest and slowest rate of adoption, with less than 20 % of the state's population covered by 2023, and no significant change in adoption rate post-Proposition 56. Additional research and evaluation time will be necessary to fully understand the long-term impact of Proposition 56 on the policies described in this report.

## CRediT authorship contribution statement

**Dennis R. Trinidad:** Writing – review & editing, Writing – original draft, Supervision, Funding acquisition. **Candice D. Donaldson:** Writing – review & editing, Writing – original draft, Validation, Data curation, Conceptualization. **Brian Dang:** Writing – review & editing, Writing – original draft, Visualization, Methodology, Formal analysis, Data curation. **Matthew D. Stone:** Writing – review & editing, Writing – original draft, Visualization, Methodology, Formal analysis, Data curation. **Thet Nwe Myo Khin:** Writing – review & editing, Writing – original draft, Project administration, Conceptualization. **Sara B. McMenamin:** Writing – review & editing, Writing – original draft, Visualization, Conceptualization. **Yuyan Shi:** Writing – review & editing, Writing – original draft, Visualization, Methodology, Conceptualization. **Tam D. Vuong:** Writing – review & editing, Writing – original draft, Validation, Methodology, Data curation. **Xueying Zhang:** Writing – review & editing, Writing – original draft, Validation, Supervision. **Karen Messer:** Writing – review & editing, Writing – original draft, Visualization, Supervision, Methodology, Conceptualization. **John P. Pierce:** Writing – review & editing, Writing – original draft, Visualization, Supervision, Methodology, Conceptualization.

## Disclaimer

The findings and conclusions in this article are those of the author(s) and do not necessarily represent the views or opinions of the California Department of Public Health or the California Health and Human Services Agency.

## Funding

This project was supported by the 10.13039/100005188Tobacco-Related Disease Research Program (TRDRP) of the 10.13039/100005595University of California, Office of the President (T31IR-1584 & T32IR-4988) and the 10.13039/100005002California Department of Public Health, California Tobacco Prevention Program (CDPH/CTPP) Contract #22–10341. The funding body had no role in the development of this paper. The content is solely the responsibility of the authors and does not necessarily represent the official views of the funding agencies.

## Declaration of competing interest

The authors declare that they have no known competing financial interests or personal relationships that could have appeared to influence the work reported in this paper.

## Data Availability

Data are publicly available
